# Circular RNA circVAPA knockdown suppresses colorectal cancer cell growth process by regulating miR-125a/CREB5 axis

**DOI:** 10.1186/s12935-020-01178-y

**Published:** 2020-03-30

**Authors:** Xiaoyu Zhang, Yingying Xu, Kenji Yamaguchi, Jinping Hu, Lianbo Zhang, Jianfeng Wang, Jifeng Tian, Wanying Chen

**Affiliations:** 1 Department of Gastrointestinal and Colorectal Surgery, China-Japan Union Hospital of Jilin University, Changchun, 130000 Jilin China; 2 Department of Ultrasound, China-Japan Union Hospital of Jilin University, Changchun, 130000 Jilin China; 3 Department of Plastic and Reconstructive Surgery, Tohoku University Graduate School, Sendai, Japan; 4 Department of Laboratory Animals, College of Animal Sciences, Jilin University, Changchun, 130062 Jilin China; 5 Department of Plastic Surgery, China-Japan Union Hospital of Jilin University, No. 126, Xiantai Street, Erdao District, Changchun, 130000 Jilin China; 6 Dapartment of Radiotherapy, China-Japan Union Hospital of Jilin University, Changchun, 130000 Jilin China

**Keywords:** CircVAPA, miR-125a, CREB5, Colorectal cancer

## Abstract

**Background:**

Colorectal cancer (CRC) is a malignant tumor, and the overall prognosis of patients with advanced CRC is still unsatisfactory. Circular RNAs (circRNAs) vesicle-associated membrane protein-associated protein A (circVAPA) could act as an underlying biomarker in CRC. This study aimed to explore the mechanism of circVAPA in the regulation of CRC growth.

**Methods:**

CircVAPA level was measured in CRC tumor tissues. The expression levels of circVAPA, VAPA mRNA, microRNA-125a (miR-125a), and cAMP response element binding 5 (CREB5) in CRC cells were detected by RT-qPCR. Cell cycle progression, migration and invasion, extracellular acidification rate (ECAR) and oxygen consumption rate (OCR) were measured by flow cytometry, transwell assays and Seahorse XF96 Glycolysis Analyzer, severally. The levels of glucose uptake, lactate and ATP production were examined by Glucose Uptake Colorimetric Assay kit, Lactate Assay kit and ATP Colorimetric Assay kit, respectively. The interaction between miR-125a and circVAPA or CREB5 was predicted by Starbase or DIANA TOOL, and verified by the dual-luciferase reporter and RNA Immunoprecipitation (RIP) assays.

**Results:**

CircVAPA level was up-regulated in CRC tumor tissues. Expression levels of circVAPA and CREB5 were increased, and miR-125a was decreased in CRC cells. CircVAPA knockdown repressed CRC cells cycle progression, migration, invasion and glycolysis. CircVAPA acted as a miR-125a sponge to regulate CREB5 expression. Rescue assay confirmed that miR-125a deletion or CREB5 overexpression weakened the inhibitory effect of circVAPA knockdown on CRC growth.

**Conclusion:**

Our studies disclosed that circVAPA knockdown suppressed CRC cells cycle progression, migration, invasion and glycolysis partly by modulating miR-125a/CREB5 axis, suggesting a potential therapeutic strategy for CRC treatment.

## Background

Globally, colorectal cancer (CRC) is a malignant and rapidly development tumor, affecting 1.8 million new cases and 881,000 deaths in 2018 [1]. In recent years, despite the substantial improvement in surgical techniques and treatment, the overall prognosis of CRC patients with advanced is still unsatisfactory [2]. Therefore, to identify the more effective therapeutic targets for CRC, it is urgent to explore the potential mechanism of tumorigenesis.

Recently, circular RNAs (circRNAs), a covalently closed-loop structure, have become a hot research area due to their important biological functions [3]. With the development of RNA sequencing technology, many circRNAs have been confirmed to show abnormal expression in various diseases, including cancer [4, 5]. For example, Han et al. reported that circBANP could exert the carcinogenic effect by boosting proliferation, migration and invasion in lung cancer development [6]. Ren et al. confirmed that circDENND4C deletion might hinder glycolysis and metastasis of breast cancer cells through binding to miR-200b/c during hypoxia [7]. In a recent document, circRNA vesicle-associated membrane protein-associated protein A (circVAPA) has been demonstrated to exert the oncogenic role by promoting proliferation in hepatocellular carcinoma [8]. Moreover, in CRC, the high expression of circVAPA was related to tumor progression and acted as an underlying biomarker [9]. It has reported that glycolysis acted as a hallmark for the progression of tumor in various cancers, including CRC [10]. Moreover, induced glycolysis and increased glucose uptake led to the promoted production of nucleotides, proteins and lipids, thereby accelerating the growth and division in tumors cells [11, 12]. However, the function of circVAPA in CRC growth including cell cycle progression and glycolysis has not been reported.

During the past decades, microRNAs (miRNAs), non-coding RNAs with about 22 nucleotides, have been proved to negatively regulate gene expression by retarding protein translation [13]. A substantial body of publications has revealed that miRNAs are involved in the pathologic processes of multiple cancers [14–16]. Previous studies have presented that miR-125a could play a tumor suppressor by regulating target genes, such as TAZ, Smurf1 and VEGFA in CRC [17–19], indicating that miR-125a is the vital role in CRC progression.

As a member of the cAMP response element (CRE)-binding protein family, CRE-binding 5 (CREB5) acted as a CRE-dependent transactivator [20]. In epithelial ovarian cancer, CREB5 facilitated cell invasion and suggested a poor prognosis [21]. Moreover, relevant literature exhibited that CREB5 could work as the tumor-promoting effect through expediting proliferation, metastasis, migration and hampering apoptosis in CRC [22].

In this manuscript, our results displayed that circVAPA knockdown impeded cell cycle progression, migration, invasion and glycolysis in CRC cells. Mechanically, circVAPA could regulate CREB5 expression by sponging miR-125a in CRC cells. This research expounded an underlying molecular mechanism of circVAPA in CRC growth process, implying a potential therapeutic strategy for CRC patients.

## Materials and methods

### Clinical samples and cell culture

Samples of CRC tumor mucosal tissues and paired normal mucosal tissues (n = 42) were provided by patients who were diagnosed with CRC at China–Japan Union Hospital of Jilin University. Each CRC patient taking part in this research signed the written informed consent and the approval was endowed by the Ethics Committee of China-Japan Union Hospital of Jilin University.

Human normal colon mucosal epithelial cell line NCM460 and CRC cell lines (HCT116 and LOVO) were collected from Cell Bank of Chinese Academy of Science (Shanghai China) and American Type Culture Collection (ATCC, Manassas, VA, USA), respectively. Cells were maintained in a humidified incubator with 5% CO_2_ at 37 °C with Roswell Park Memorial Institute 1640 medium (RPMI 1640, Transgene, Beijing, China), when RPMI 1640 cultured cells were added with 10% fetal bovine serum (FBS, Gibco, Carlsbad, CA, USA).

### RNA extraction and real-time quantitative polymerase chain reaction (RT-qPCR)

Total RNA was extracted from CRC tissues and cells using TRIzol reagent (Invitrogen, Carlsbad, CA, USA), followed by incubation at 37 °C for 20 min with or without RNase R (Epicentre, Shanghai, China). After purification with phenol–chloroform (Solarbio, Beijing, China), total RNA was reversely transcribed into the complementary DNA (cDNA) by a PrimeScript™ RT Reagent Kit (Takara, Dalian, China) in accordance with the supplier’s direction. Whereafter, with the help of SYBR^®^ Premix Ex Taq™ reagent (TaKaRa), RT-qPCR was conducted on a Roche Light Cycler 480 Real-time PCR Amplifier following the operation manual. The relative levels of genes were calculated by the 2^− ΔΔCt^ method, normalizing with house-keeping gene glyceraldehyde-3-phosphate dehydrogenase (GAPDH) or U6 small nuclear RNA. The primers used were presented as follows:

CircVAPA: 5′-TGGATTCCAAATTGAGATGCGTATT-3′ (sense), 5′-CACTTTTCTATCCGATGGATTTCGC-3′ (antisense); miR-125a: 5′- GGTCATTCCCTGAGACCCTTTAAC -3′ (sense), 5′-GTGCAGGGTCCGAGGT-3′ (antisense); CREB5: 5′-AGATGGTCCTCTGTTGGGAA-3′ (sense), 5′-TGGACACGGTTATGAGAATGA-3′ (antisense); VAPA mRNA: 5′-GAAGCTGTGTGGAAAGAGGC-3′ (sense), 5′-GAGCATTCC. CTGGTGGAGTT-3′ (antisense); GAPDH: 5′-GTCAACGGATTTGGTCTGTATT-3′ (sense), 5′-AGTCTTCTGGGTGGCAGTGAT-3′ (antisense); U6: 5′-GCTCGCTTCGGCAGCACA-3′ (sense), 5′-GAGGTATTCGCACCAGAGGA-3′ (antisense).

### Cell transfection

CircVAPA small interfering RNA (si-circVAPA), si-CREB5 and their negative control (si-con), miR-125a mimics (miR-125a), miR-125a inhibitor (anti-miR-125a) and their negative controls (miR-con and anti-miR-con) were provided by GenePharma (Shanghai, China). Moreover, pcDNA3.1-circVAPA (circVAPA), pcDNA3.1-CREB5 (CREB5) and pcDNA 3.1 empty vector (vector) were obtained from Genecreat (Wuhan, China). The oligonucleotide and vector were transfected in HCT116 and LOVO cells using Lipofectamine 3000 reagents (Invitrogen)based on the instruction guidelines.

### Cell cycle assay

Simply, transfected CRC cells were fixed with ice ethanol, followed by treatment with RNase A (Takara) and Propidium Iodide (PI) (Invitrogen). At 24 h after incubation, a FACSCalibur (Becton–Dickinson, Franklin Lakes, NJ, USA) was employed to detect the DNA content. Percentage of cell-cycle stages was analyzed with ModFit LT (Verity Software House, Topsham, ME, USA).

### Cell migration and invasion assays

The abilities of migration and invasion were examined with Transwell chambers (Corning Incorporated, Corning, NY, USA) and matrigel (BD Biosciences, San Jose, CA, USA). In the assay, cells were seeded on the upper chambers with (for invasion assay) or without matrigel (BD Biosciences; for migration assay). As a chemoattractant, the medium containing FBS (Gibco) was added into the lower chambers. At 48 h after transfection, the cells on the lower surface were fixed and stained, and the cells remaining in the upper chamber were removed with cotton swabs. The staining cells were imaged and counted under an inverted microscope.

### Extracellular acidification rate (ECAR) and oxygen consumption rate (OCR) assays

For this assay, ECAR was measured on Seahorse XF96 Glycolysis Analyzer (Seahorse Bioscience, North Billerica, MA, USA) with Seahorse XF Glycolysis Stress Test Kit (Seahorse Bioscience) based on the user’s guidebook. In short, transfected cells were plated into a Seahorse XF 96 cell culture microplate, followed by the measurements of baseline. At the indicated time points, Glucose, Oligomycin (oxidative phosphorylation inhibitor) and 2-DG (glycolytic inhibitor) were sequentially injected. Similarly, OCR was detected with Seahorse XF Cell Mito Stress Test Kit (Seahorse Bioscience) on this Analyzer. Oligomycin, FCCP (reversible inhibitor of oxidative phosphorylation) and Rote + AA (mitochondrial complex I inhibitor rotenone plus the mitochondrial complex III inhibitor antimycin A) were injected. Finally, the results were analyzed with the Seahorse XF96 Wave software (Seahorse Bioscience).

### Glucose uptake, lactate and ATP assay

Glucose uptake, lactate and ATP production in CRC cells were examined by Glucose Uptake Colorimetric Assay kit (Biovision, Milpitas, CA, USA), Lactate Assay kit (Sigma St. Louis, MO, USA) and ATP Colorimetric Assay kit (Sigma), severally, in line with the supplier’s direction.

### Dual-luciferase reporter assay

Briefly, the sequences of circVAPA or CREB5 3′ UTR containing putative binding sites of wild-type miR-125a were amplified and inserted into the luciferase report pGL3 vector (Promega, Madison, WI, USA), obtaining circVAPA-wild type (WT) or CREB5-WT reporter vector. Subsequently, the constructed reporter vector was transfected with miR-125a, miR-125a + vector, and miR-125a + CREB5 or miR-125a + circVAPA into HCT116 or LOVO cells using Lipofectamine 3000 reagents (Invitrogen). At 48 h after transfection, the luciferase activity was assessed with the dual-luciferase reporter assay kit (Promega).

### RNA immunoprecipitation (RIP) assay

RIP assay was implemented with a Magna RIP kit (Millipore, Bedford, MA, USA) as per the manufacturer’s protocol. In this assay, transfected CRC cells were harvested and lysed in complete RIP lysis buffer. Whereafter, magnetic beads conjugated with anti-Argonaute2 (Ago2, Millipore) or anti-immunoglobulin G (IgG, Millipore) were added into the buffer. And then, proteinase K was applied to separate the RNA–protein complexes from beads. At last, the levels of circVAPA, miR-125a and CREB5 in the precipitates were detected with RT-qPCR.

### Western blot assay

In short, radioimmunoprecipitation lysis buffer (RIPA; Beyotime, Ningbo, China) was utilized to harvest the lysates from CRC tissues and cells, followed by the separation with 10% sodium dodecyl sulfate–polyacrylamide gel electrophoresis (SDS-PAGE). Then, the proteins were transferred to a nitrocellulose membrane (Millipore), and blocked with 5% non-fat milk. After the incubation with specific primary antibody: CREB5 (1:1000, ab168928, Abcam, Cambridge, MA, USA) and β-actin (1:1000, ab8226, Abcam), the secondary antibody was probed with the membrane. Finally, protein signals were visualized with enhanced chemiluminescence (ECL, Amersham Pharmacia Biotech, Piscataway, NJ, USA).

### Statistical analysis

All values were presented as mean ± standard deviation (SD) at three independent assays. Statistical analysis was calculated with SPSS 20.0 software. Differences between groups were performed with Student’s *t-* test or one-way analysis of variance (ANOVA). *P* values of less than 0.05 were recognized as statistically significant.

## Results

### CircVAPA was up-regulated in CRC tissues and cells

**Fig. 1:**
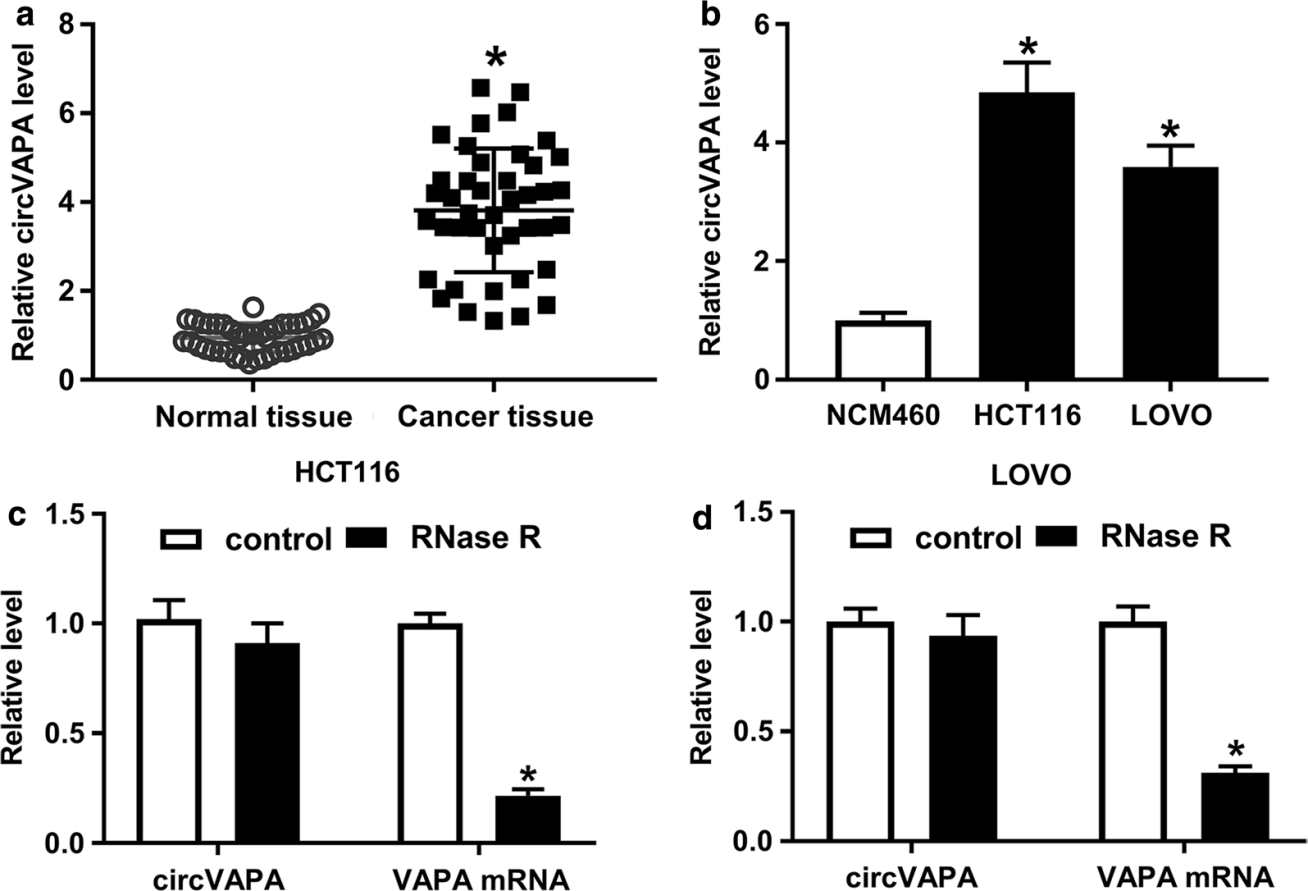
CircVAPA was elevated in CRC tissues and cells. **a** RT-qPCR assay was applied to measure the expression level of circVAPA in 42 pairs of CRC tumor mucosal tissues and adjacent normal mucosal tissues. **b** CircVAPA expression level was examined in CRC cell lines (HCT116 and LOVO) and human normal colon mucosal epithelial cell line (NCM460). **c**, **d** Relative levels of circVAPA and VAPA mRNA were tested in HCT116 and LOVO cells treated with or without RNase R. *P < 0.05

To investigate the function of circVAPA in CRC, its expression level was detected by using RT-qPCR. Compared with 42 normal mucosal tissues, circVAPA level was highly expressed in 42 CRC tumor mucosal tissues (Fig. 1a). Then, we further confirmed that circVAPA level was evidently increased in CRC cell lines (HCT116 and LOVO) relative to human normal colon mucosal epithelial cell line NCM460 (Fig. 1b). Moreover, to verify that circVAPA was a circular RNA, HCT116 and LOVO cells were treated with RNase R. As displayed in Fig. 1c, d RNase R treatment effectively downregulated VAPA mRNA level in HCT116 and LOVO cells, but not that of circVAPA expression level, suggesting that circVAPA was resistant to RNase R digestion. In a word, as a circular RNA, circVAPA might be involved in CRC progression.

### CircVAPA knockdown suppressed cycle progression, migration and invasion in CRC cells

**Fig. 2:**
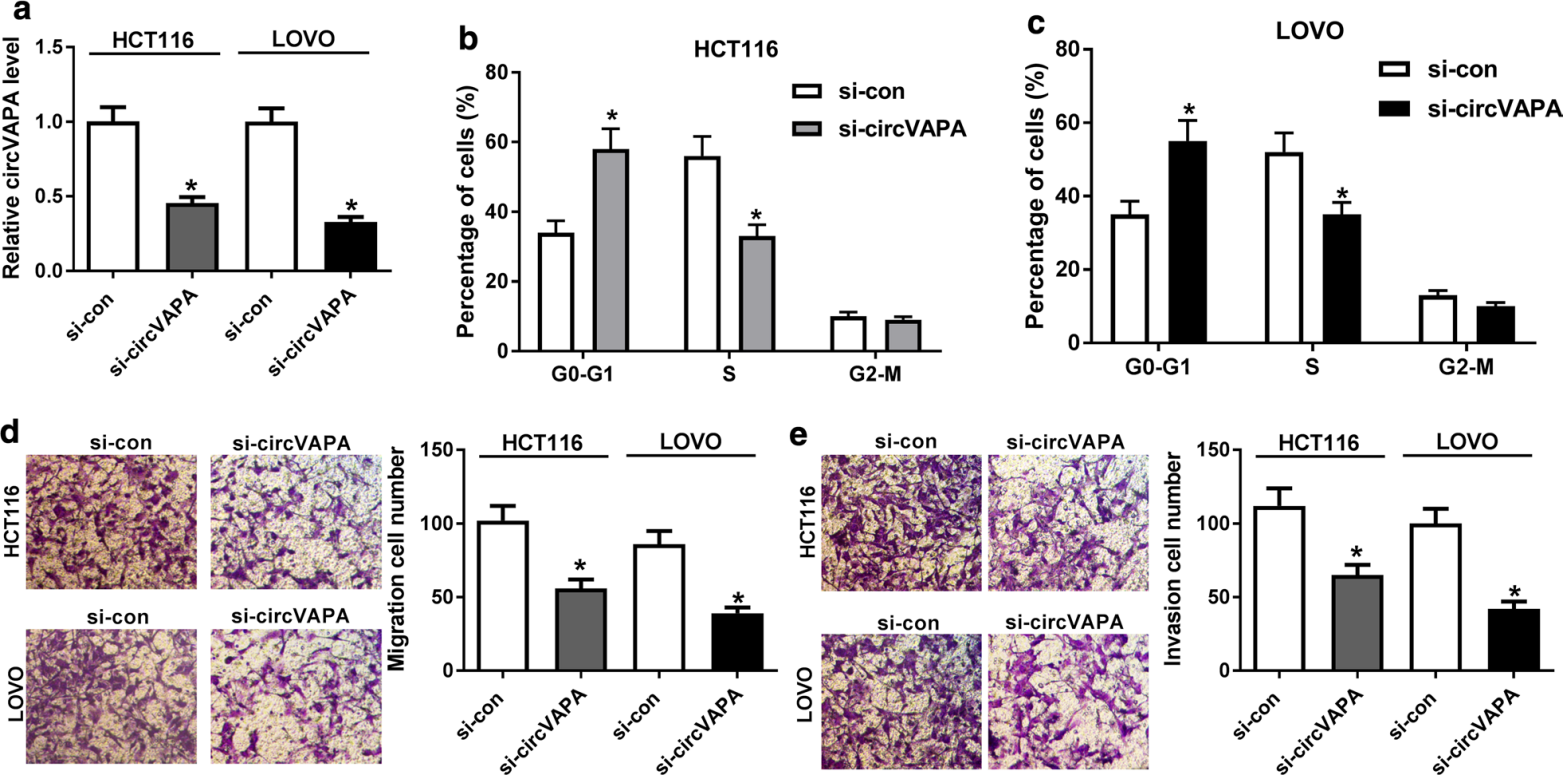
CircVAPA deficiency repressed cycle progression, migration and invasion of CRC cells. **a**Transfection efficiency of si-circVAPA in HCT116 and LOVO cells was detected by RT-qPCR assay. **b, c** Flow cytometry was applied to test cell cycle progression of G0/G1 phase in si-circVAPA-transfected HCT116 and LOVO cells. **d**, **e** Transwell assay was conduct to detect the abilities of migratory and invasive in si-circVAPA-transfected HCT116 and LOVO cells. *P < 0.05

Considering the high expression of circVAPA in CRC, we knocked down circVAPA expression in HCT116 and LOVO cells. As shown in Fig. 2a, transfection of si-circVAPA obviously reduced circVAPA expression level, manifesting that si-circVAPA could be used for the subsequent loss-of-function assays. Flow cytometry assay exhibited that circVAPA deletion inhibited cell cycle progression in HCT116 and LOVO cells (Fig. 2b, c). Meanwhile, decreased migration and invasion were viewed caused by the downregulation of circVAPA (Fig. 2d, e). Overall, circVAPA silencing could hinder cell cycle progression, migration and invasion of CRC cells.

### CircVAPA knockdown repressed glycolysis of CRC cells

**Fig. 3:**
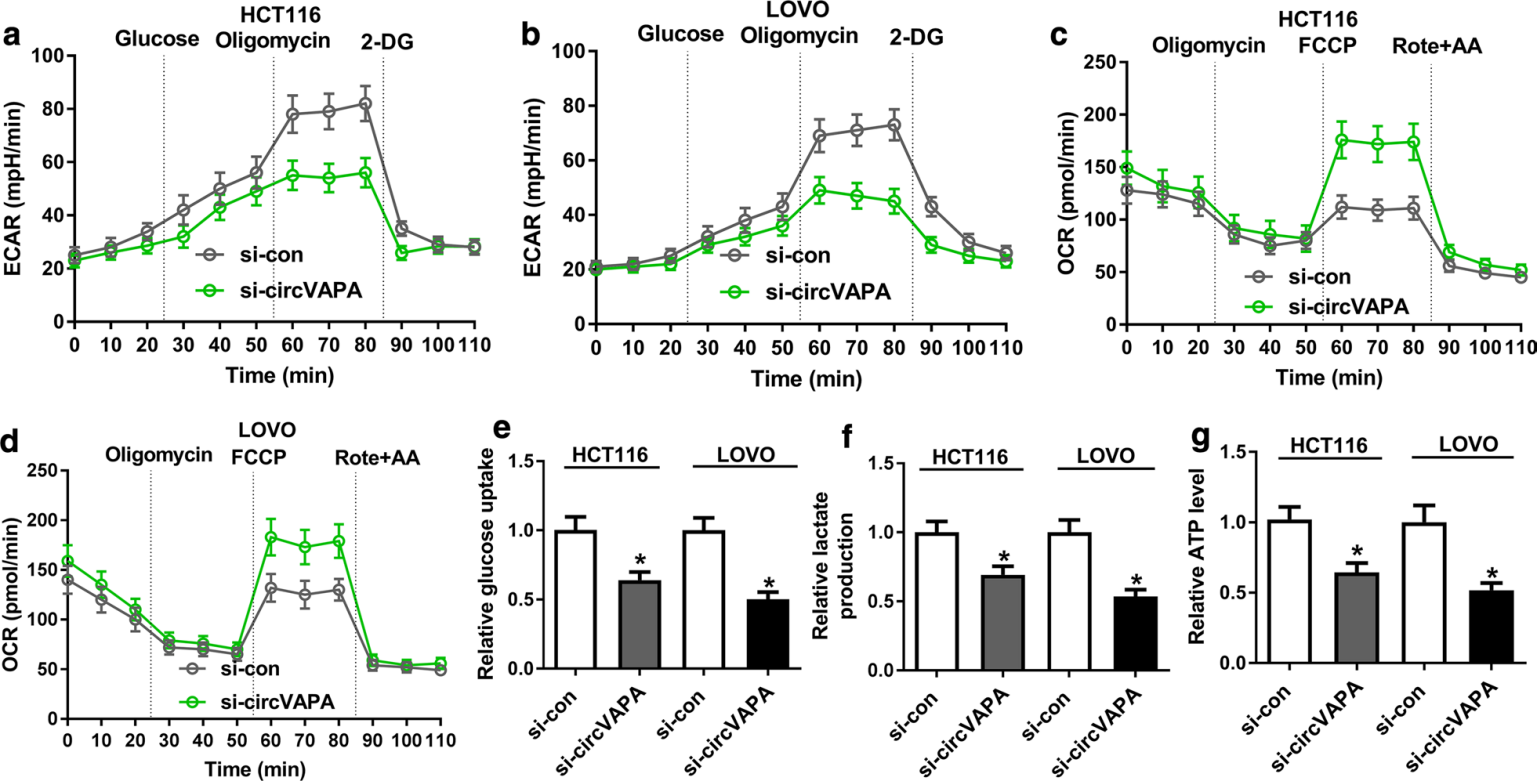
CircVAPA knockdown blocked aerobic glycolysis of CRC cells. **a, b** Extracellular acid ratio (ECAR) upon cell was examined by Seahorse XF Glycolysis Stress Test Kit in si-circVAPA-transfected HCT116 and LOVOcells. **c**, **d** Oxygen consumption rate (OCR) was measured by Seahorse XF Cell Mito Stress Test Kit in si-circVAPA-transfected HCT116 and LOVO cells. **e**–**g** The levels of glucose uptake, lactate production and ATP were detected in si-circVAPA-transfected HCT116 and LOVO cells. *P < 0.05

Glycolysis has been reported to exert the positive role in the progression of CRC [23], hence, we explored whether circVAPA has a function in glycolysis in CRC cells. Meanwhile, extracellular acidification rate (ECAR), a product of glycolysis, was largely determined by lactic acid release. The ECAR approximated the glycolysis flux rate [24], and oxygen consumption rate (OCR) was an indicator of mitochondrial oxidative respiration. Therefore, with the treatment of glucose, oligomycin or 2-DG, we detected the effect of circVAPA knockdown on the levels of ECAR and OCR in CRC cells. As illustrated in Fig. 3a, b circVAPA deletion led to a conspicuous decrease in ECAR in HCT116 and LOVO cells. Moreover, accelerated OCR (Fig. 3c, d) and the reduced level of glucose uptagke (Fig. 3e), lactate production (Fig. 3f) and ATP (Fig. 3g) in HCT116 and LOVO cells with si-circVAPA were viewed. Collectively, circVAPA deficiency impeded CRC cells glycolysis.

### CircVAPA regulated CREB5 expression by sponging miR-125a

**Fig. 4:**
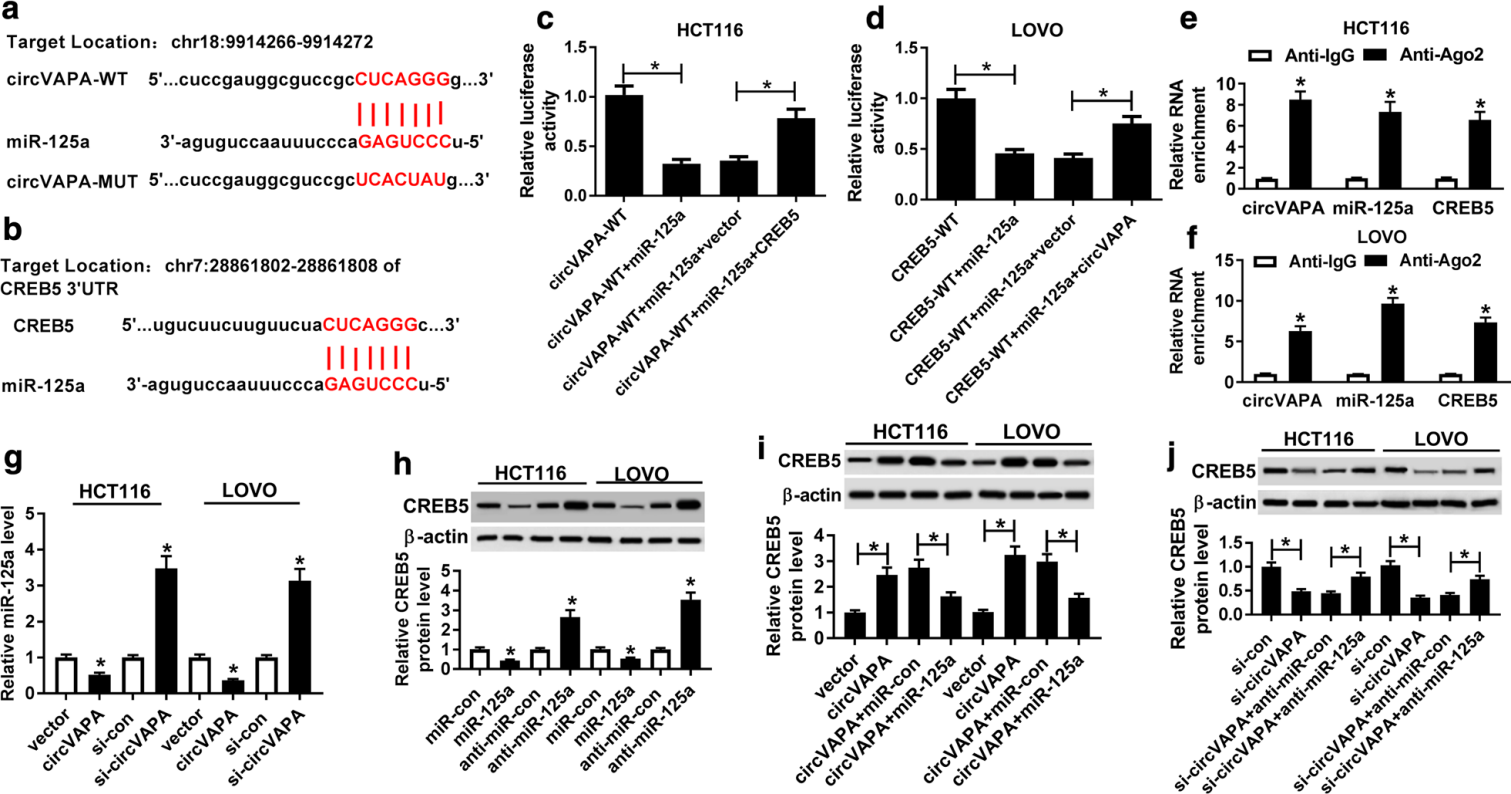
CREB5 was positively regulated by circVAPA/miR-125a. **a**, **b** The binding sites betweenmiR-125a and circVAPA or CREB5 3′UTR were predicted by starBase or DIANA TOOL. **c**, **d** Dual-luciferase reporter assay was performed to confirm the bindings among circVAPA, miR-125a and CREB5. **e**, **f** The interacting between circVAPA, miR-125a and CREB5 was proved by RIP assay. **g** The effects of circVAPA upregulation and deletion on miR-125a expression were assessed in HCT116 and LOVO cells. **h** The effects of miR-125a overexpression and knockdown on CREB5 protein level were detected in HCT116 and LOVO cells. **i** The protein levels of CREB5 were examined in HCT116 and LOVO cells transfected with vector, circVAPA, circVAPA + miR-con and circVAPA + miR-125a. **j** CREB5 protein levels were measured in HCT116 and LOVO cells transfected with si-con, si-circVAPA, si-circVAPA + anti-miR-con and si-circVAPA + anti-miR-125a. *P < 0.05

Given that circVAPA exerted the crucial role in the cell cycle, migration, invasion and glycolysis of CRC cells. Meanwhile, a substantial body of recent research has disclosed that circRNA could serve as miRNA sponge to regulate mRNA expression [25]. Therefore, through bioinformatics analysis, there was a potential binding between miR-125a and circVAPA or CREB5 3′UTR (Fig. 4a, b). Whereafter, we used the dual-luciferase reporter assay to further verify the prediction results in HCT116 and LOVO cells. Data suggested that the miR-125a notably dampened the luciferase activity of circVAPA-WT reporter vector, while re-introduction of CREB5 3′UTR effectively abrogated the effect in HCT116 cells (Fig. 4c). Meanwhile, our results also indicated that miR-125a could obviously reduced the luciferase activity of CREB5 WT reporter vector, which was abrogated by circVAPA upregulation in LOVO cells (Fig. 4d). Consistent with bioinformatics analysis and dual-luciferase reporter assay, RIP assay indicated that the levels of circVAPA, miR-125a and CREB5 were strikingly enriched in Ago2 pellets of HCT116 and LOVO cell extracts compared with normal IgG control group (Fig. 4e, f). Besides, miR-125a level was evidently reduced in HCT116 and LOVO cells transfected with circVAPA, while its level was notably increased in CRC cells transfected with si-circVAPA (Fig. 4g). Meanwhile, we found that miR-125a overexpression repressed the level of CREB5 in HCT116 and LOVO cells, and miR-125a downregulation promoted CREB5 level (Fig. 4h). Intriguingly, in HCT116 and LOVO cells, miR-125a upregulation strikingly impaired the promotion effect of circVAPA overexpression on CREB5 protein level, on the contrary, miR-125a inhibition remarkably overturned the negative effect of si-circVAPA on CREB5 protein level (Fig. 4i, j). All of these suggested that circVAPA could perform as a sponge of miR-125a to positively modulate CREB5 expression.

### CircVAPA regulated cell cycle progression, migration and invasion by miR-125a/CREB5 axis in CRC cells

**Fig. 5:**
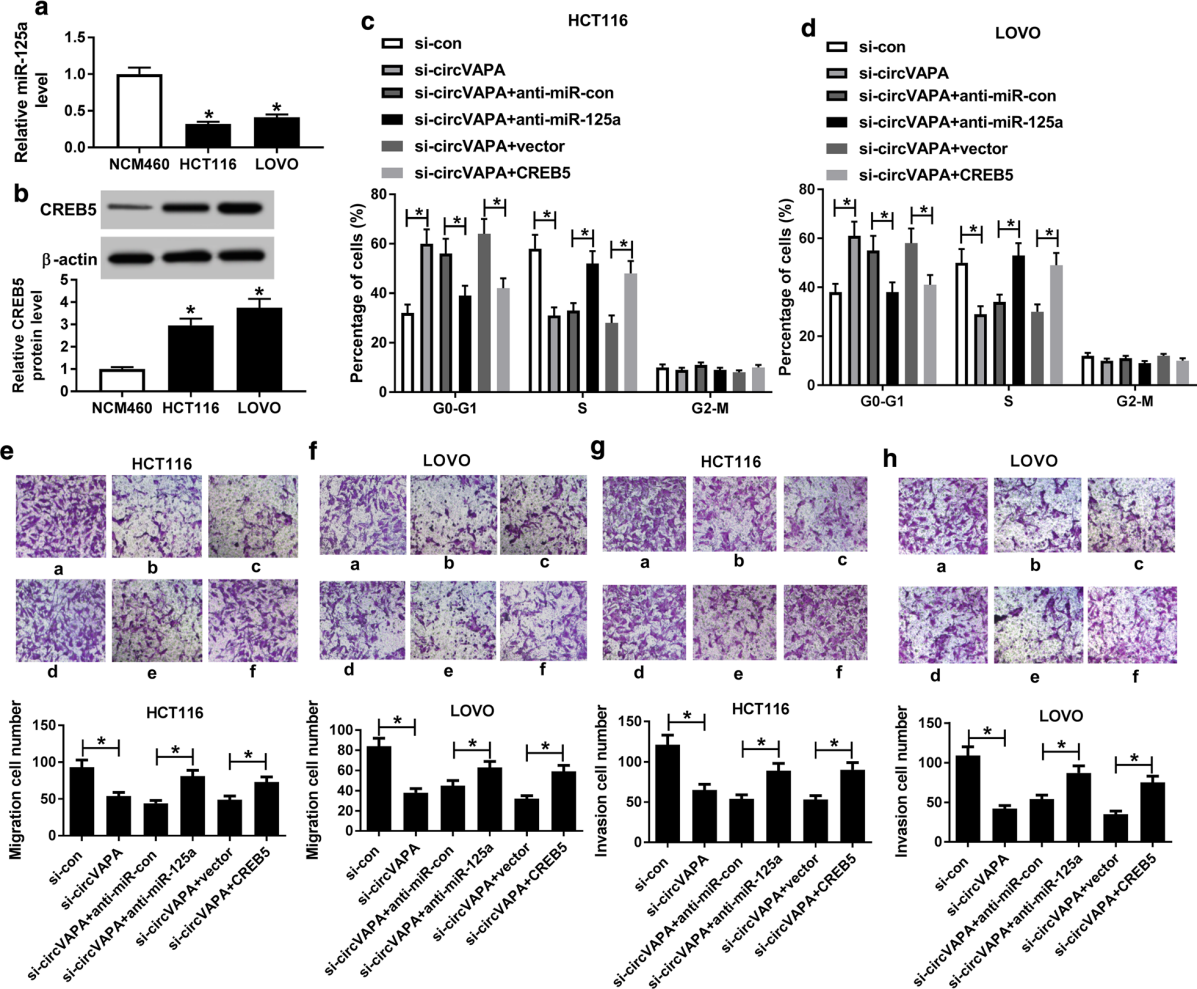
Regulation of circVAPA on cell cycle progression, migration and invasion was mediated by miR-125a/CREB5 axis. **a**, **b** The expression levels of miR-125a and CREB5 were detected in CRC cell lines (HCT116 and LOVO) and human normal colon mucosal epithelial cell line (NCM460). **c**, **d** Cell cycle in G0/G1 phase was examined in HCT116 and LOVOcells transfected with si-con, si-circVAPA, si-circVAPA + anti-miR-con, si-circVAPA + anti-miR-125a, si-circVAPA + vector and si-circVAPA + CREB5. **e**, **f** Migratory ability was assessed in transfected HCT116 and LOVO cells. **g**, **h** Invasive capacity was tested in transfected HCT116 and LOVO cells. *P < 0.05

As mentioned above, we speculated that circVAPA could exert its function through miR-125a/CREB5 axis in CRC. Simultaneously, low expression of miR-125a and high expression of CREB5 were observed in HCT116 and LOVO cells relative to NCM460 (Fig. 5a, b). Thus, to prove the inference, we executed the rescue assay in HCT116 and LOVO cells. As presented in Fig. 5c, d cell cycle arrested by circVAPA silencing was partly abrogated by miR-125a knockdown or CREB5 overexpression in HCT116 and LOVO cells. Transwell analysis result testified that the suppression of migration and invasion triggered by circVAPA knockdown was abolished through anti-miR-125a or CREB5 in HCT116 and LOVO cells (Fig. 5e–h). In addition, our data suggested that CREB5 upregulation boosted cell cycle progression, migration, invasion and glycolysis in HCT116 and LOVO cells, however, the knockdown of CREB5 produced the opposite results (Additional file 1: Figure S1). All the data described above indicated that circVAPA could regulate cycle progression, migration and invasion through miR-125a/CREB5 axis in CRC cells.

### Regulation of circVAPA on glycolysis was mediated by miR-125a/CREB5 axis

**Fig. 6:**
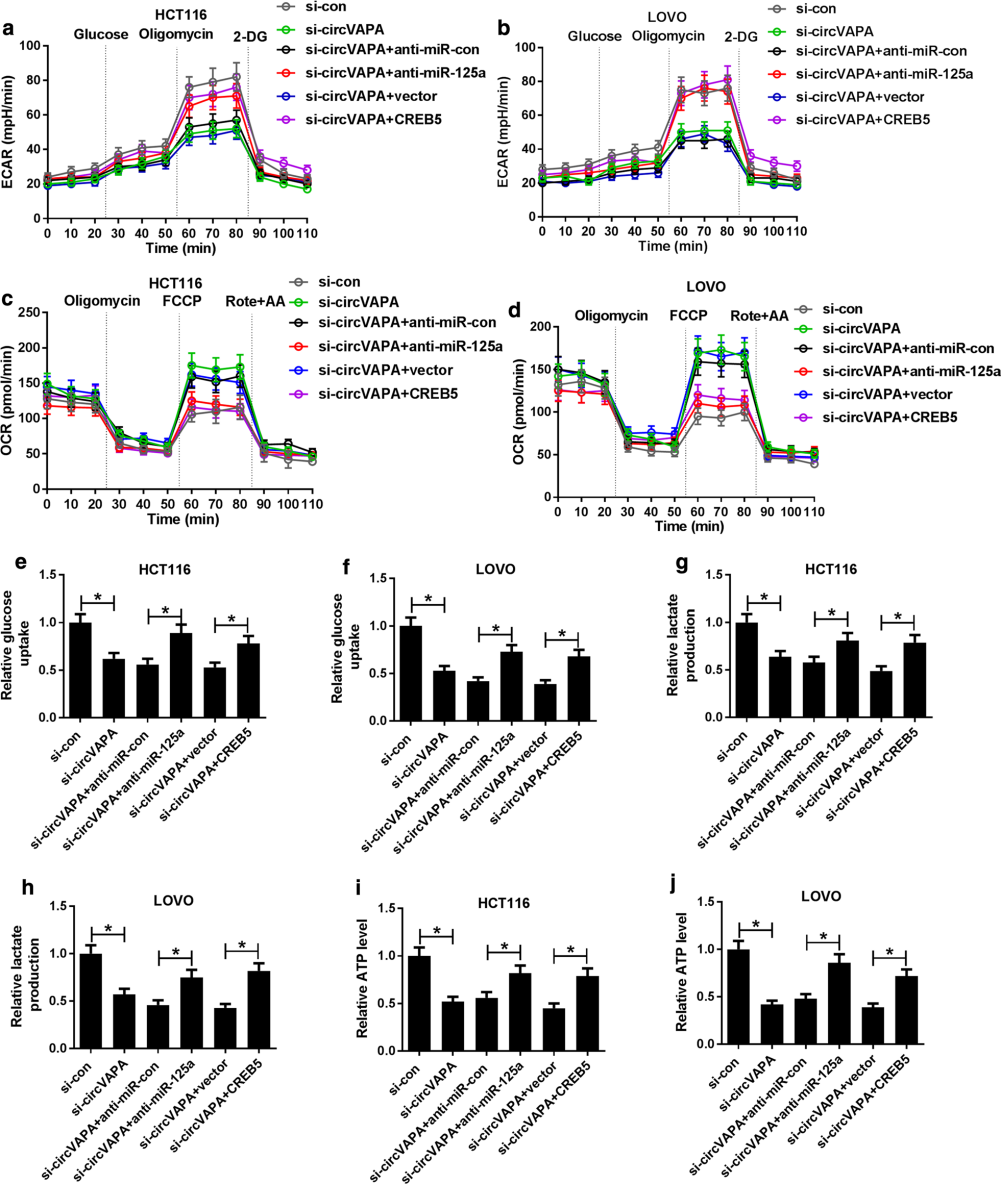
CircVAPA-mediated glycolysis was regulated by miR-125a/CREB5 axis. **a**, **b** The change of ECAR level was measured in HCT116 and LOVOcells transfected with si-con, si-circVAPA, si-circVAPA + anti-miR-con, si-circVAPA + anti-miR-125a, si-circVAPA + vector and si-circVAPA + CREB5. **c**, **d** The change of OCR was detected in transfected HCT116 and LOVO cells. **e**, **f** Glucose uptake was examined in transfected HCT116 and LOVO cells. **g**, **h** The production of lactate was detected in transfected HCT116 and LOVO cells. **i**, **j** ATP was determined in transfected HCT116 and LOVO cells. *P < 0.05

Next, we further identify whether circVAPA could modulate glycolysis by miR-125a/CREB5 axis in CRC cells. As exhibited in Fig. 6a, b miR-125a knockdown or CREB5 overexpression significantly mitigated the inhibitory effect of circVAPA deletion on ECAR in HCT116 and LOVO cells. Additionally, we also found that anti-miR-125a or CREB5 obviously abolished circVAPA silencing-mediated increase in OCR (Fig. 6c, d), and decline in the levels of glucose uptake (Fig. 6e, f), lactate production (Fig. 6g, h) and ATP (Fig. 6i, j) in HCT116 and LOVO cells. Taken together, these results suggested that circVAPA knockdown could exert the inhibitory effect on glycolysis through miR-125a/CREB5 axis in CRC cells.

## Discussion

In recent years, accumulating evidence has indicated that circRNAs could be defined as remarkable prognostic biomarkers of diverse cancers [26], such as epithelial ovarian cancer [27], hepatocellular carcinoma [28] and CRC [29]. Indeed, as the research moves along, circRNAs have been verified to be actively involved in the formation and development of CRC. For instance, circHIPK3 was an indicator of CRC prognosis, and excess of circHIPK3 was confirmed to elevate cell growth and metastasis through binding to miR-7 [30]. Consistently, circ_0020397 could exert the carcinogenic role in CRC to reinforce cell viability and invasion, weaken apoptosis by sponging miR-138 to modulate TERT and PD-L1 expression [31]. Notably, a relevant report has presented that circVAPA, a novel circRNA, was upregulated in CRC, and circVAPA was implicated with tumorigenesis, such as proliferation, migration and apoptosis [9]. Nevertheless, in other aspects of CRC growth, including cell cycle and glycolysis, the function and mechanism of circVAPA required further exploration.

In this paper, circVAPA was identified as highly expressed in CRC tissues and cells relative to their respective control groups, suggesting the underlying poor prognosis. Apart from that, RNase R treatment reduced the mRNA level of VAPA, while it had little effect on the circVAPA level. In other words, compared with the linear RNA, circRNA was more suitable as an underlying biomarker due to the structural stability. Functionally, the downregulation of circVAPA repressed cell cycle progression, migration, invasion and glycolysis of CRC cells, supporting the inhibitory action in CRC tumor growth.

As is widely recognized, circRNAs could interact with miRNAs to modulate mRNA [32–34]. Thus, in this manuscript, we further explored whether circVAPA could exert its role in CRC through regulating the miRNA/mRNA axis. Bioinformatics predicts results indicated there were binding seeds between miR-125a and circVAPA or CREB5, as confirmed by dual-luciferase reporter and RIP assays. Intriguingly, miR-125a level was negatively correlated with circVAPA or CREB5 in CRC cells. Importantly, we also found that circVAPA regulated CREB5 expression level by targeting miR-125a. That was to say, we first demonstrated that circVAPA influence CREB5 expression by sponging miR-125a in CRC cells.

Additionally, recent studies showed that the abnormal expressions of miR-125a and CREB5 were involved in the development of CRC [17, 22, 35]. Thus, in this manuscript, we further explored whether circVAPA silencing could exert the anti-tumor effect in CRC cells through modulating miR-125a/CREB5 axis. Rescue assays verified that miR-125a silencing or CREB5 overexpression could undermine the suppressive function of circVAPA knockdown on cell cycle progression, migration, invasion and glycolysis in CRC cells, further validating that circVAPA knockdown hindered CRC growth through miR-125a/CREB5 axis. The inhibitory action of miR-125a on glycolysis was also reported in laryngeal squamous cell carcinoma and thyroid cancer [36, 37].

In the future research, the downstream molecular mechanism of circVAPA/miR-125a/CREB5 axis will continue to be explored.

## Conclusion

To sum up, our study presented that circVAPA acted as miR-125a sponge to affect CREB5 expression, thereby regulating CRC growth process, contributing to explore the new underlying therapeutic target for CRC.

## Supplementary information

**Additional file 1: Figure S1.** The effects of CREB5 overexpression or knockdown on cell cycle progression, migration, invasion and glycolysis were detected in CRC cells. *P < 0.05.

## Data Availability

The datasets used and/or analysed during the current study are available from the corresponding author on reasonable request.

## Abbreviations

CRC: Colorectal cancer
CREB5: CAMP response element binding 5
ECAR: Extracellular acidification rate
OCR: Oxygen con-sumption rate
RIP: RNA immunoprecipitation
