# Supplementary figures and images for: Circular RNA circVAPA knockdown suppresses colorectal cancer cell growth process by regulating miR-125a/CREB5 axis
Source: Cancer Cell Int. 2020 Mar 30;20:103. doi: 10.1186/s12935-020-01178-y (PMC7106619; doi:10.1186/s12935-020-01178-y)

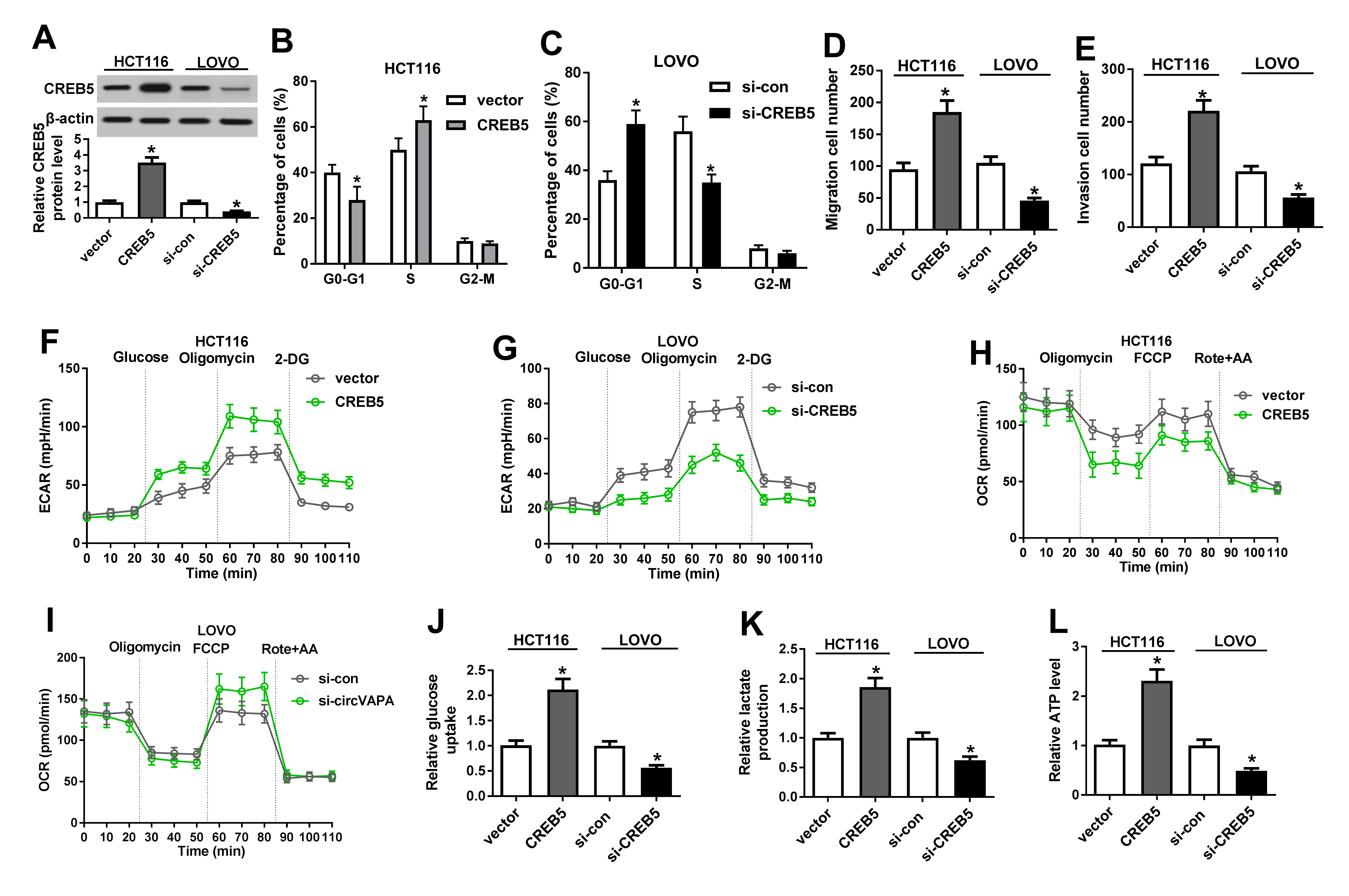

Supplement: Supplementary file 1 — Additional file 1: Figure S1. The effects of CREB5 overexpression or knockdown on cell cycle progression, migration, invasion and glycolysis were detected in CRC cells. *P < 0.05. [file 12935_2020_1178_MOESM1_ESM.tif]
